# The study of tryptophol containing emulgel on fungal reduction and skin irritation

**DOI:** 10.1038/s41598-023-46121-z

**Published:** 2023-11-02

**Authors:** Thitinan Kitisin, Watcharamat Muangkaew, Natthawut Thitipramote, Arnon Pudgerd, Passanesh Sukphopetch

**Affiliations:** 1 Department of Microbiology and Immunology, Faculty of Tropical Medicine, Mahidol University, Bangkok, Thailand; 2 School of Cosmetic Science, Mae Fah Luang University, Chiang Rai, Thailand; 3 Division of Anatomy, School of Medical Science, University of Phayao, Muang, Phayao, Thailand

**Keywords:** Drug discovery, Microbiology, Medical research

## Abstract

Tryptophol (TOH), a fungal quorum-sensing molecule, that possesses anti-fungal activities for controlling the growth of human pathogenic fungi. In the present study, we developed TOH-containing emulgel formulations and examined the antifungal activities and potential use as topical treatments on the skin. The results showed that TOH-containing emulgel at 1000 μM has excellent physical characteristics as homogenous, stability, and inhibits the growth of 30 species of human pathogenic fungi *in vitro*. TOH-containing emulgel did not cause skin irritation in mouse model of irritation and in healthy human volunteers. Moreover, an increase in skin hydration and a decrease in trans-epidermal water loss (TEWL) were observed after TOH-containing emulgel treatment on human skin. Our findings indicated that TOH-containing emulgel can be utilize as an antifungal agent for topical treatment against fungal infections on the skin.

## Introduction

Fungi can communicate to their extracellular environments by producing and releasing their small diffusible chemical signaling molecules known as fungal quorum-sensing molecules (QSM)^1^. Fungal QSMs have been shown to have a variety of biological activities such as cell-to-cell communication, morphogenesis, biofilm formation, and virulence, which ultimately controls the growth of another fungi^2^. To date, they are 4 main groups of QSMs including farnesol (FOH), tyrosol (TYR), phenylethanol (PE), and tryptophol (TOH)^3^.

Tryptophol (C_10_H_11_NO, TOH; molecular weight, 161.20) is a QSM that can be isolated from yeasts such as *Candida albicans* and *Saccharomyces cerevisiae*^4^. Our previous studies have demonstrated that TOH can inhibit the growth of *Candida albicans*^5^ and *Scedosporium apiospermum*^4^. Thus, TOH has a high potential to be developed as an innovative product containing active antifungal agent for the treatment of fungal infections.

To develop a product containing TOH, vehicles that are used to incorporate TOH for dermal delivery are greatly concerns^6^. Repeated topical application of TOH may adversely cause skin irritation and contact dermatitis^7^. Emulsion-based formulations are currently the chosen formulation for topical delivery of active agents, which show good interaction on the skin^8^.

The aim of the study was to investigate the antifungal effects TOH-containing emulgel *in vitro* and skin irritation *in vivo* as well as on healthy human volunteers. Moreover, the cosmeceutical benefits of TOH-containing emulgel was also investigated.

## Results

### Formulation and evaluation of TOH-containing emulgel

With the aim to develop the TOH-containing emulgel from emulgel base recipe, the emulgel prototype products containing 100 μM and 1000 μM of TOH were successfully developed. The physical and chemical properties of TOH-containing emulgel were evaluated previously^9^. Moreover, an emulgel containing 1000 μM of voriconazole (VOZ) was also produced. The parameters of emulgel base, TOH- and VOZ-containing emulgel were presented in Table 1. The manufactured 100 μM and 1000 μM of TOH- and 1000 μM of VOZ- containing emulgel appeared as white, translucent, homogenous, and non-greasy. However, high concentrations of active compounds (TOH and VOZ) caused an increase in viscosity and reduced the spread ability of the products (Table 1). There was no phase separation found both all manufactured emulgel formulations. There were no color changes or physical instability involving phase separation or inconsistency increases. Nevertheless, the pH of the manufactured emulgel formulations were found to be in the acceptable range of 4.5-5.5.

**Table 1:** Physical properties of different optimized emulgel formulations.

Properties	Emulgel formulation			
	Emulgel base	100 µM TOH	1000 µM TOH	1000 µM VOZ
Color	White and translucent	White and translucent	White and translucent	White and creamy
Homogeneity	Excellent	Excellent	Excellent	Excellent
Consistency	Excellent	Excellent	Excellent	Excellent
Phase separation	None	None	None	None
Spread ability (gm.cm/sec)	25.00 ± 0.50^a^	24.50 ± 0.50^b^	22.00 ± 0.50^c^	14.00 ± 1.20^d^
Viscosity (cp)	2,350 ± 40^a^	2,806 ± 55^b^	8,970 ± 50^c^	11,380 ± 100^d^
Centrifugation test	Excellent	Excellent	Excellent	Excellent
Cooling-heating and freeze-thaw cycle test	Excellent	Excellent	Excellent	Excellent
pH	5.37 ± 0.01^a^	5.52 ± 0.01^b^	5.55 ± 0.01^b^	4.50 ± 0.05^c^

Values are expressed as mean ± standard deviation (SD).

*TOH* Tryptophol, *VOZ* voriconazole.

^a–d^Indicated *P* < 0.05 compared between experimental conditions by one-way ANOVA followed by Tukey’s multiple comparison test.

### Antifungal susceptibility of TOH-containing emulgel

*In-Vitro* antifungal activities of manufactured TOH-containing emulgel formulations were evaluated in 30 strains of fungal pathogens using the agar spot assay. Firstly, the colonies diameters of azole-resistant/or azole-sensitive strains including *Scedosporium apiospermum* (CBS 117410), *S. boydii* (CBS 120157), *Lomentospora prolificans* (CM324)^9^, *Aspergillus fumigatus* (CL-262)^10^, *A. fumigatus* (AF293), *Candida albicans* (ATCC 96901), and *C. albicans* (ATCC 90028) were determined after 14 days of incubation with emulgel base, 100 μM and 1000 μM of TOH- and 1000 μM of VOZ-containing emulgel (Table 2). Moreover, the results showed that 1000 μM of TOH-containing emulgel remarkedly lessened the growth rate of these azole-resistant strains than 100 μM of TOH- and 1000 μM of VOZ-containing emulgel. The colonies diameters of common fungal skin pathogens were further investigated. In the group of hyaline molds, 1000 μM of TOH-containing emulgel remarkedly lessened the growth rate of *Trichophyton mentagrophytes* (ATCC MYA-4439), *T. rubrum* (ATCC MYA-4438), *T. verrucosum* (ATCC 42898), *T. tonsurans*^11^, *Microsporum canis*^11^, *M. gypseum*^12^, and *Epidermophyton floccosum* (ATCC 52066) than 100 μM of TOH- and 1000 μM of VOZ-containing emulgel. In the group of dematiaceous molds, 1000 μM of TOH-containing emulgel remarkedly lessened the growth rate of *A. recifei* (ATCC 36328), *Fonsecaea pedrosoi, E. dermatitidis* (ATCC 76204), and *Bipolaris maydis* than 100 μM of TOH- and 1000 μM of VOZ-containing emulgel. Moreover, 1000 μM of TOH-containing emulgel remarkedly lessened the growth rate of dimorphic fungi (*Sporothrix schenckii* (ATCC 58251) and *Talaromyces marneffei*^13^) than 100 μM of TOH- and 1000 μM of VOZ-containing emulgel. In pathogenic yeasts, 1000 μM of TOH-containing emulgel remarkedly lessened the growth rate of *Malassezia furfur* (ATCC 12078), *T. asahii* (ATCC 90039), *C. tropicalis, C. dubliniensis* (ATCC MYA-646), *Cryptococcus neoformans* (ATCC 32045), *C. gattii* (ATCC 56992), *Lodderomyces elongisporus* (ATCC 11503), *Mucor* spp.^13^, *Rhizopus* spp., and *Beauveria bassiana* (ATCC 74040) than 100 μM of TOH- and 1000 μM of VOZ-containing emulgel. Thus, the effective concentration of TOH-containing emulgel at 1000 μM was chosen due to its board antifungal activities.

**Table 2:** Colony diameters of tryptophol (TOH)-containing emulgels treated fungal pathogens after incubation at 37 °C for 14 days.

Fungal strains	Colony diameter at Day 14 in mm (mean ± SD)		
	100 µM TOH emulgel	1000 µM TOH emulgel	1000 µM VOZ emulgel
*A. fumigatus* (AF293)	No growth	No growth	No growth
*A. fumigatus* (CL-262)	7.50 ± 1.050^b^	No growth^a^	8.00 ± 0.95^b^
*A. recifei* (ATCC 36328)	8.50 ± 1.05^b^	No growth^a^	11.50 ± 2.45^b^
*B. bassiana* (ATCC 74040)	24.50 ± 0.70^b^	19.50 ± 2.15^a^	27.50 ± 1.50^c^
*B. maydis*	14.50 ± 1.05^b^	3.50 ± 0.95^a^	18.50 ± 3.40^b^
*C. albicans* (ATCC 90028)	No growth	No growth	No growth
*C. albicans* (ATCC 96901)	No growth	No growth	No growth
*C. dubliniensis* (ATCC MYA-646)	No growth	No growth	No growth
*C. gattii* (ATCC 56992)	No growth	No growth	7.00 ± 1.50^b^
*C. neoformans* (ATCC 32045)	No growth	No growth	4.50 ± 2.10^b^
*C. tropicalis*	No growth	No growth	No growth
*E. dermatitidis* (ATCC 76204)	9.00 ± 2.80^b^	No growth^a^	9.50 ± 2.75^b^
*E. floccosum* (ATCC 52066)	3.50 ± 1.50^b^	No growth^a^	9.50 ± 1.00^c^
*F. pedrosoi*	12.50 ± 3.15^b^	No growth^a^	10.50 ± 3.55^b^
*L. elongisporus* (ATCC 11503)	No growth	No growth	2.50 ± 0.90^b^
*L. prolificans* (CM324)	11.50 ± 1.00^c^	No growth^a^	8.50 ± 1.15^b^
*M. canis*	No growth	No growth	No growth
*M. furfur* (ATCC 12078)	No growth	No growth	No growth
*M. gypseum*	No growth	No growth	No growth
*Mucor* spp.	No growth	No growth	No growth
*Rhizopus* spp.	No growth	No growth	No growth
*S. apiospermum* (CBS 117410)	No growth	No growth	No growth
*S. boydii* (CBS 120157)	No growth	No growth	No growth
*S. schenckii* (ATCC 58251)	No growth^a^	No growth^a^	3.00 ± 1.95^b^
*T. asahii* (ATCC 90039)	No growth	No growth	No growth
*T. marneffei*	8.00 ± 0.85^a^	10.00 ± 1.00^b^	11.50 ± 2.45^b^
*T. mentagrophytes* (ATCC MYA-4439)	No growth	No growth	No growth
*T. rubrum* (ATCC MYA-4438)	No growth	No growth	No growth
*T. tonsurans*	No growth	No growth	No growth
*T. verrucosum*	6.50 ± 0.75^b^	No growth^a^	8.00 ± 0.65^b^

*TOH* Tryptophol, *VOZ* voriconazole.

^a–c^Indicates statistically significant *P*<0.05 in each experimental condition by two-way ANOVA.

### In vitro human foreskin fibroblast cell viability, and cytotoxicity

**Figure 1:**
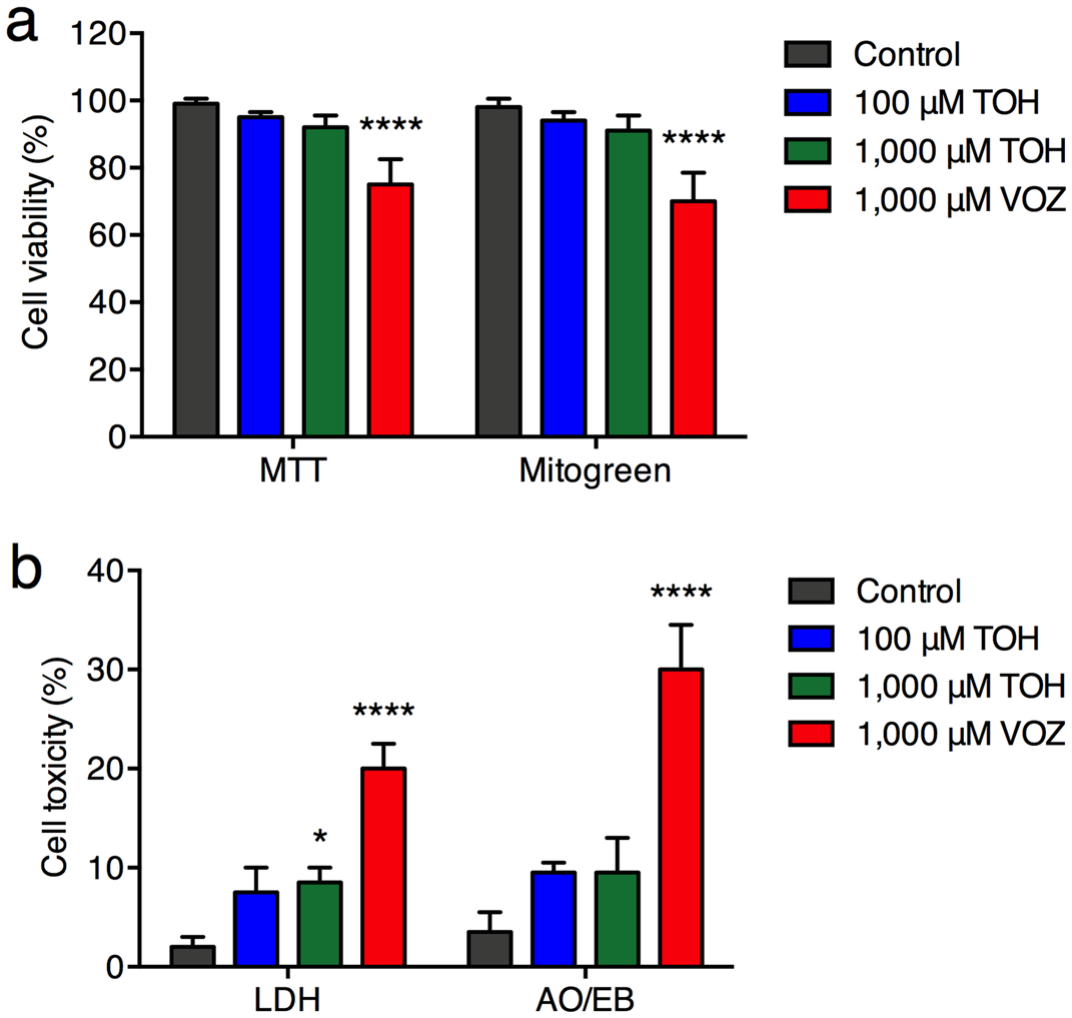
Cell viability (**a**) and cytotoxicity (**b**) of TOH-treated human foreskin fibroblasts (HFF- 1) cells at 37 °C as determined by MTT, Mitogreen, LDH, and dual AO/EB staining assays. Percentages of cell viability and cytotoxicity were shown as mean ± SD. *P < 0.05 and ****P < 0.0001 indicate significant differences compared with the control group (concentration 0). *TOH* Tryptophol, *VOZ* voriconazole.

To further determine the potential use as topical TOH-containing emulgel, human foreskin fibroblasts (HFF-1) cell viability and cytotoxicity were investigated after incubating for 24 h with 100 μM and 1000 μM of TOH and 1000 μM of VOZ using MTT (3-(4,5- dimethylthiazol-2-yl)-2,5-diphenyl tetrazolium bromide), MitoGreen, lactate dehydrogenase (LDH), and dual Acridine Orange/Ethidium Bromide AO/EB staining assays. The results showed that HFF-1 cell viability did not show a significant change after treating with 100 and 1000 μM of TOH as determined by MTT and MitoGreen assays (Fig. 1a). However, HFF-1 cell viability (75 ± 7.50% and 70 ± 8.50%, respectively) was significantly decreased at 1000 μM of VOZ when compared to controls. Moreover, incubation of 100 and 1000 μM of TOH did not induce HFF-1 cell cytotoxicity as determined by LDH and dual AO/EB staining assays, whereas, 1000 μM of VOZ significantly increased HFF-1 cell cytotoxicity (20 ± 2.50% and 30 ± 4.50%, respectively) when compared to controls (Fig 1b). Thus, the results suggested that TOH at 1000 μM exhibits antifungal activates against common skin pathogenic fungi without compromising the health of human skin *in vitro.*

### Evaluation of TOH-containing emulgel on mouse skin irritation

**Figure 2:**
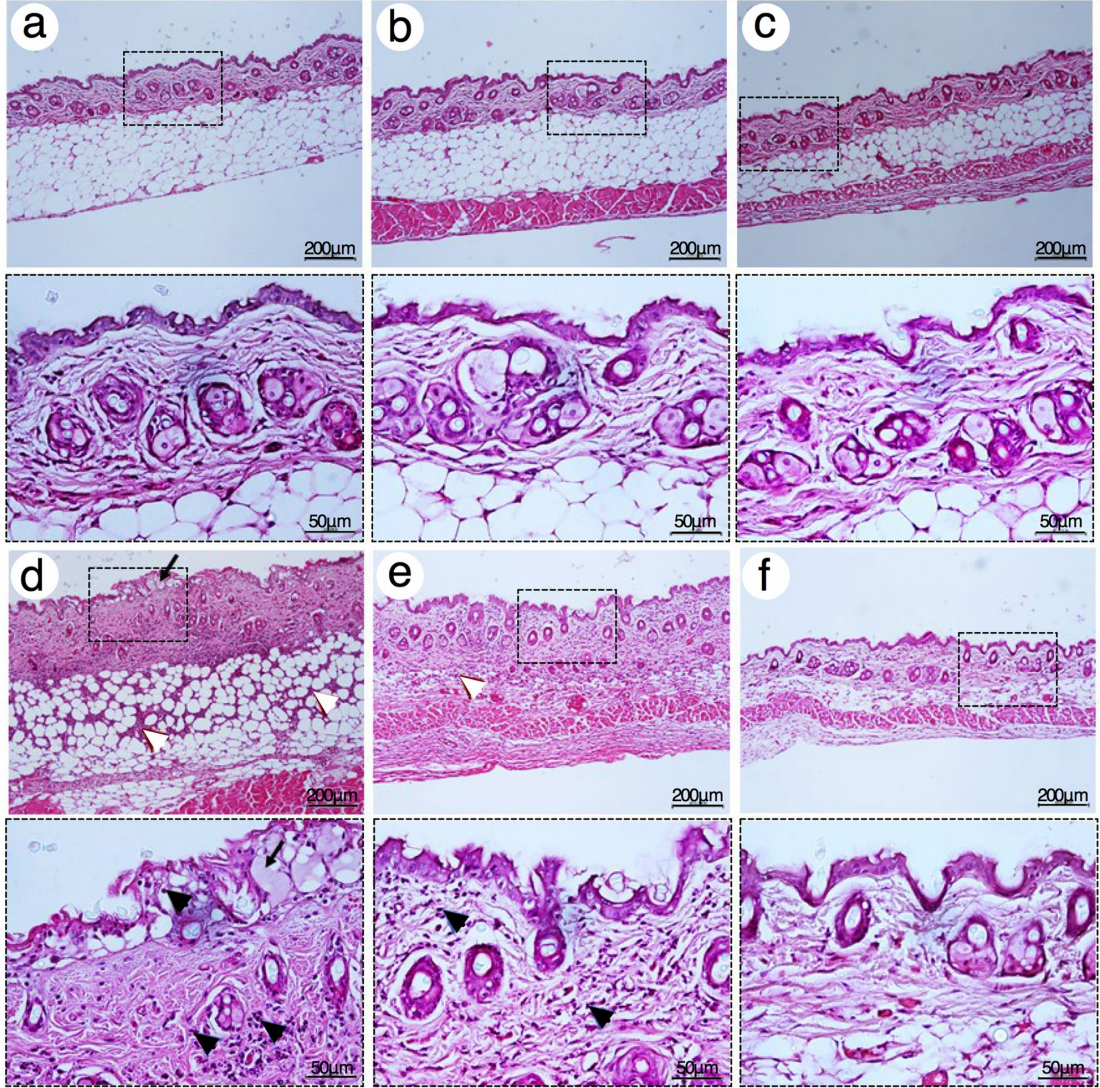
Representative H&E photographs of mouse skin after 24 h post-topical application with 1000 μM TOH-containing emulgel. (**a**) Emulgel cream base, (**b**) Commercial shielding cream, (**c**) 1000 μM VOZ emulgel, (**d**) croton oil, (**e**) 5% SLS, and (**f**) 1000 μM TOH emulgel. Arrows indicated the spongiosis at epidermis. Dash boxes magnified each specific Black and White arrowheads indicated inflammatory cells infiltration at the epidermis and dermis, respectively. Scale bar = 200 μm and = 50 μm.

Single irritation test on mouse skin was used examined an acute irritant contact dermatitis (AICD) of 1000 μM TOH-containing emulgel. After 24 h post-topical application, croton oil and 5% Sodium Lauryl Sulfate (SLS) were able to induce mild to moderate edema with loss of skin creases, whereas, emulgel cream base, commercial shielding cream, 1000 μM VOZ-containing emulgel and 1000 μM TOH-containing emulgel did not induce any skin changes as determined by visual assessment. The notable histological features of acute irritant contact dermatitis were spongiosis, accumulation of fluid in the epidermis, and inflammatory cells infiltrations in epidermis, dermis, and subcutaneous tissues in the group treated with croton oil (Fig. 2d) and lessened in the group treated with 5% SLS (Fig. 2e). No histological characteristics of AICD were found in the group treated with emulgel cream base (Fig. 2a), commercial shielding cream (Fig. 2b), 1000 μM VOZ-containing emulgel (Fig. 2c), and 1000 μM TOH-containing emulgel (Fig. 2f).

**Figure 3:**
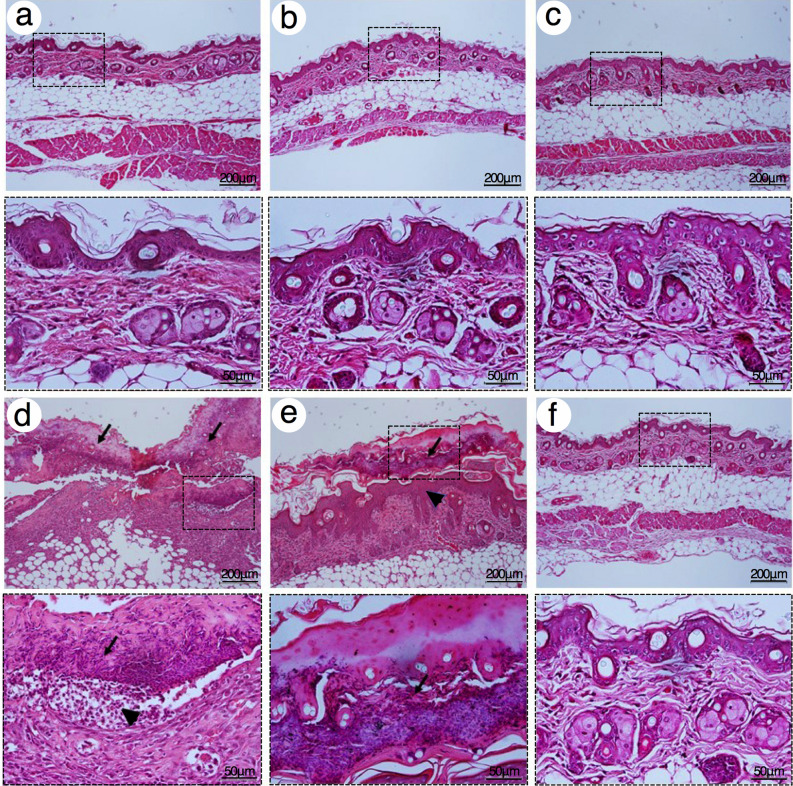
Representative H&E photographs of mouse skin after 7 days of topical application with 1000 μM TOH-containing emulgel. (**a**) Emulgel cream base, (**b**) Commercial shielding cream, (**c**) 1000 μM VOZ emulgel, (**d**) croton oil, (**e**) 5% SLS, and (**f**) 1000 μM TOH emulgel. Arrows indicated the spongiosis at epidermis. Dash boxes magnified each specific Black arrowheads indicated inflammatory cells infiltration at the epidermis. Scale bar = 200 μm and = 50 μm.

**Figure 4:**
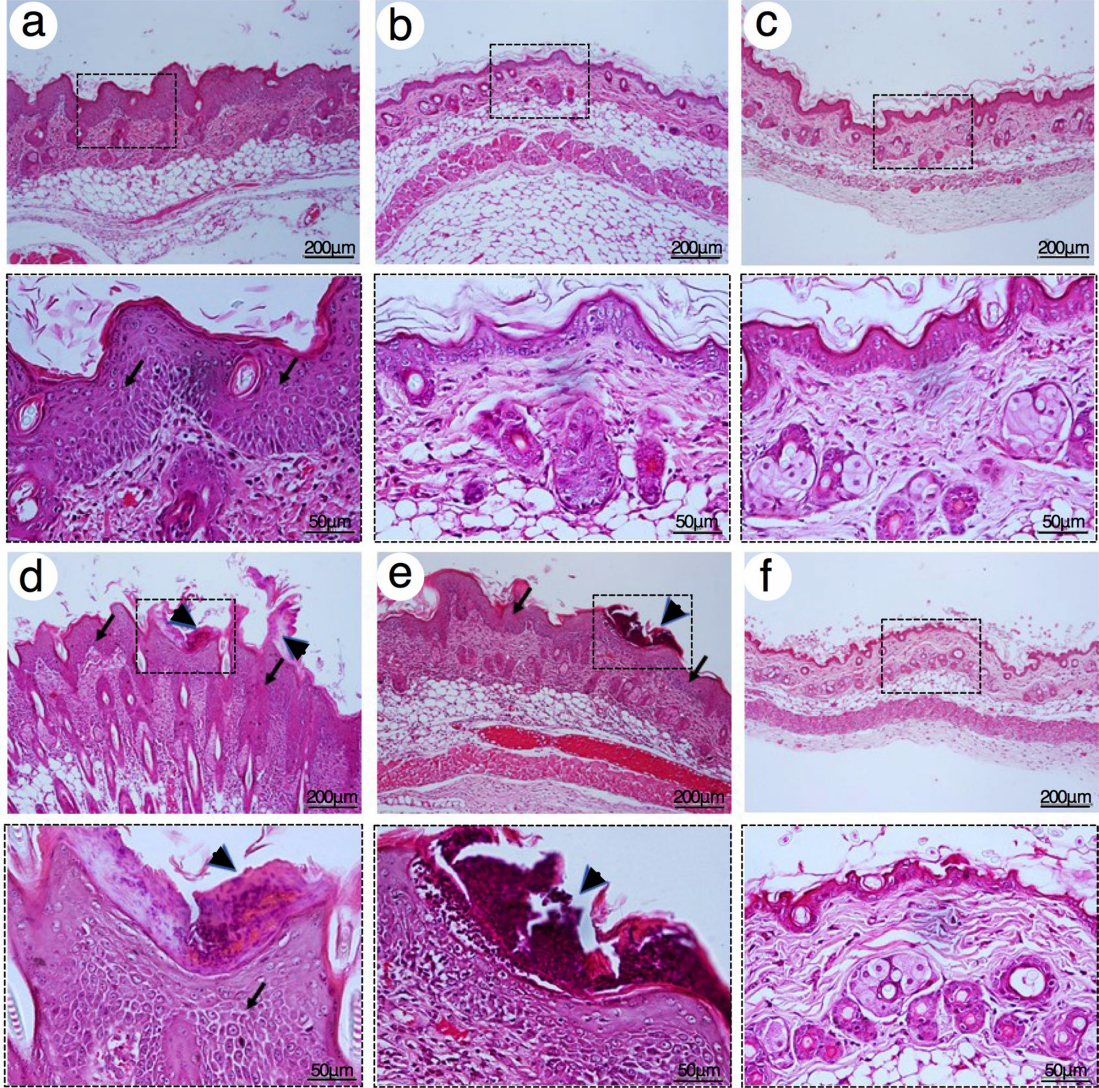
Representative H&E photographs of mouse skin after 14 days of topical application with 1000 μM TOH-containing emulgel. (**a**) Emulgel cream base, (**b**) Commercial shielding cream, (**c**) 1000 μM VOZ emulgel, (**d**) croton oil, (**e**) 5% SLS, and (**f**) 1000 μM TOH emulgel. Arrows indicated the spongiosis at epidermis. Dash boxes magnified each specific Black arrowheads indicated inflammatory cells infiltration at the epidermis. Scale bar = 200 μm and = 50 μm.

Cumulative irritation test on mouse skin was used examined a chronic irritant contact dermatitis (CICD) of 1000 μM TOH-containing emulgel. Severe epidermal hyperplasia, spongiosis, inflammatory cells infiltrations, and dilatation of dermal vessels were found in the group treated with croton oil after 7 days (Fig. 3d) and 14 days of application (Fig. 4d) and lessened in the group treated with 5% SLS after 7 days (Fig. 3e) and 14 days of application (Fig. 4e). No histological characteristics of CICD were found in the group treated with emulgel cream base (Figs. 3a and 4a), commercial shielding cream (Figs. 3b and 4b), 1000 μM VOZ-containing emulgel (Figs. 3c and 4c), and 1000 μM TOH-containing emulgel (Figs. 3f and 4f) after 7 days and 14 days of application, respectively. Therefore, the present study suggested that 1000 μM TOH-containing emulgel did not induce neither acute or chronic irritation *in vivo*.

### Evaluation of TOH-containing emulgel on human skin irritation

**Figure 5:**
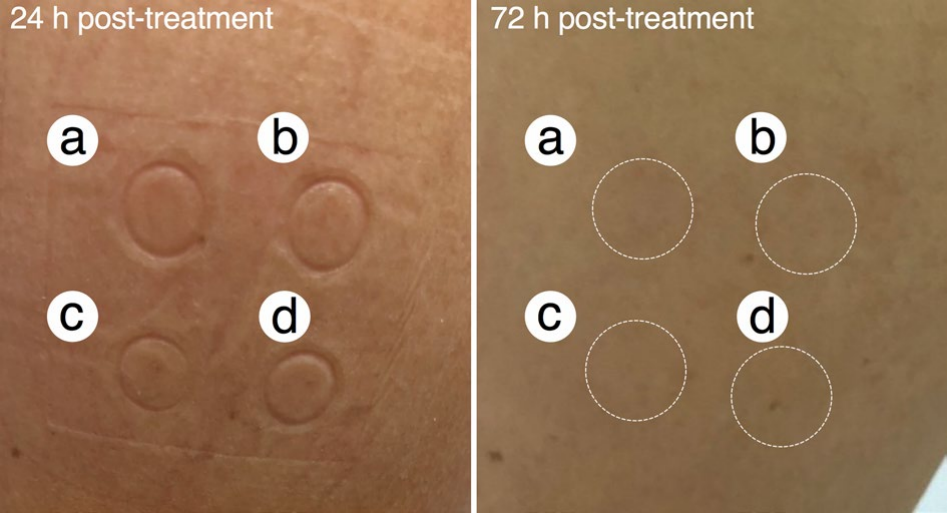
Representative photographs of skin reactions to, (**a**) commercial shielding cream, (**b**) 1000 μM TOH-containing emulgel, (**c**) 1000 μM VOZ-containing emulgel, and (**d**) emulgel cream base after 24 h post- and 72 h post-topical application.

Skin irritation test was determined in thirty healthy volunteers after topical treatment of 1000 μM TOH-containing emulgel. After 24 h post-topical application, no visual characteristics of skin irritation or dermatitis observed in the group treated with commercial shielding cream (Fig. 5a), 1000 μM TOH-containing emulgel (Fig. 5b), 1000 μM VOZ-containing emulgel (Fig. 5c), and emulgel cream base (Fig. 5d). Moreover, no skin irritation was observed after 72 h post-topical application in all treatments (Fig. 5). Therefore, the present study suggested that 1000 μM TOH-containing emulgel treatment did not cause skin irritation in human volunteers.

### Evaluation of TOH-containing emulgel on human skin physiological properties

**Figure 6:**
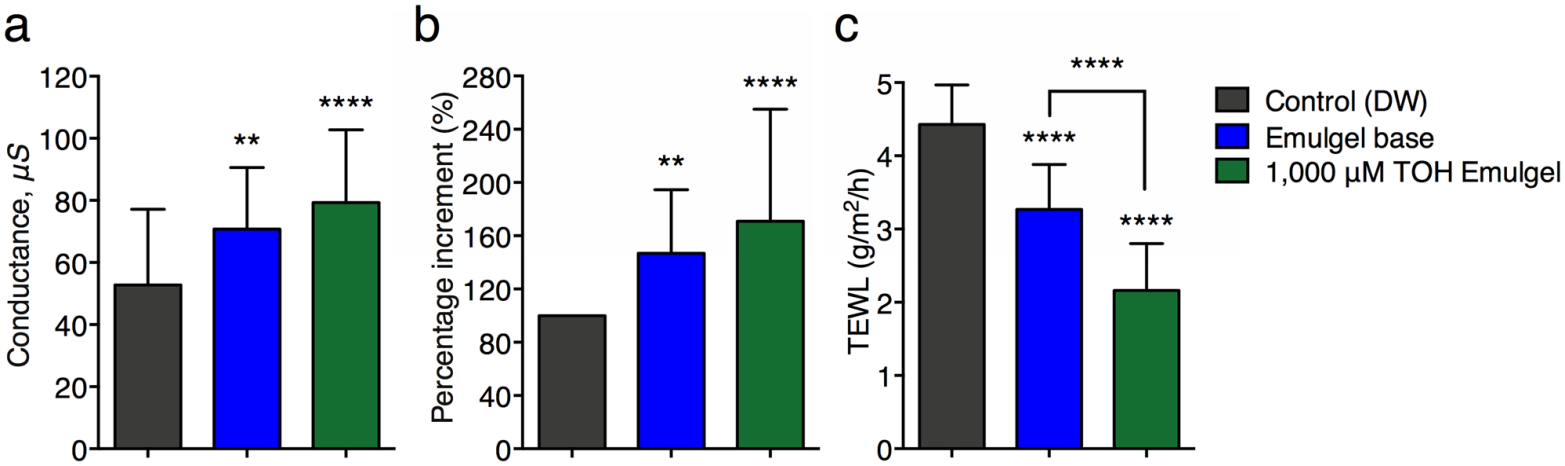
Noninvasive assessments for (**a**) skin hydration, (**b**) percentage of hydration changes, and **C** TEWL readings at 14 days post-application of 1000 μM TOH-containing emulgel (n = 30). Data were shown as mean ± SD. **P < 0.01 and ****P < 0.0001 indicate significant differences compared with the control group (concentration 0). *TOH* Tryptophol.

Hydration values measured on the skin of volunteers treated with 1000 μM TOH-containing emulgel are significantly higher than emulgel base and distilled water treated areas (Fig. 6a). Skin hydration was significantly increased after treated with 1000 μM TOH-containing emulgel (Fig. 6b). Moreover, treatment of 1000 μM TOH-containing emulgel significantly reduced transepidermal water loss (TEWL) than other products (Fig. 6c). Therefore, the present study suggested that 1000 μM TOH-containing emulgel treatment enhances other cosmeceutical benefits to the human skin.

## Discussion

Superficial mycosis is caused by pathogenic fungi that affects superficial layer of the skin^14^. Several species of human pathogenic fungi can infect deeper than the superficial layer of the skin causing cutaneous mycosis^15^. These skin mycoses are common worldwide, which in some cases, causing cosmetically poor appearance with severe inflammation^16^. Antifungal drug such as voriconazole (VOZ), a triazole compound, is the drug-of-choice antifungal agent against fungal infections^17^. However, fungal infections that resistant to triazoles such as VOZ has become a concern with increases cases over the past decade^18^. Thus, the present study was conducted and developed to evaluate the effectiveness of alternative topical treatment to treat various pathogenic fungi as a tryptophol (TOH)-containing emulgel.

In general, TOH can be naturally found as a quorum sensing molecule (QSM) in seed plants, bacteria, and fungi^19^. TOH affects fungi morphogenesis, which provides a potential use as an antifungal agent various fungal pathogen species^20^. We have previously demonstrated that *in vitro* treatment of TOH suppresses the pathogenicity of *C. albicans*. In addition, treatment of TOH was found to induce *C. albicans* apoptosis via the transcriptional upregulation of caspase recruitment domain-containing protein *(CARD)-9* and *Noxa* and the downregulation of *Bcl-2*^5^. We also previously demonstrated that treatment of TOH at 100 μM reduces the germ tube length and biofilm formation of *S. apiospermum in vitro*. Moreover, TOH-containing emulgel was developed at 100 μM, which reduced *S. apiospermum*-induced eumycetoma formation *in vivo*^9^. Also the antifungal activity of TOH both *in vitro* and *in vivo* have been extensively studied, whether antifungal activity and safety of TOH-containing emulgel for clinical use remains to be elucidated.

In the present study, we demonstrated that TOH at 100 μM and 1000 μM was successfully incorporated into emulgel base containing SEPIGEL 305^9^. Moreover, TOH-containing emulgel in the present study exhibited excellent in cosmeceutical levels including homogeneity, consistency, and stability with pH range of 5.5, which compromised with the pH of the skin^21^. Interestingly, we further demonstrated that 1000 μM TOH-containing emulgel exhibited excellent in antifungal activity against 30 pathogenic fungi including azole-resistant strains, hyaline and dematiaceous molds, dimorphic fungi, and yeasts than 1000 μM VOZ-containing emulgel *in vitro*. Thus, our study suggested that the manufactured TOH-containing emulgel at 1000 μM was optimal for topical use *in vivo* with antifungal activity against wide ranges of human cutaneous pathogenic fungi.

In this report, we demonstrated that TOH caused less cytotoxic to human foreskin fibroblast (HFF-1) cells than VOZ *in vitro* suggesting that TOH is safe to use as topical treatment *in vivo*. We further determined the safety use of TOH-containing emulgel in human volunteers by determining both acute irritant contact dermatitis (AICD) and chronic irritant contact dermatitis (CICD) caused by 1000 μM TOH-containing emulgel prior to commercialize the product. From the present study, our results suggested that either single or repeated exposure of 1000 μM TOH-containing emulgel does not cause skin irritation or any histological changes, which suitable for long term use^22^.

No skin irritation was observed in human volunteers after 1000 μM TOH-containing emulgel treatments. Moreover, skin hydration and less water loss were gained after 1000 μM TOH-containing emulgel treatments. Therefore, our present study showed that 1000 μM TOH-containing emulgel is suitable to be used as an alternative antifungal product for topical treatment. Hence, it is possible to infer that the 1000 μM TOH-containing emulgel was safe for topical application on human skin without skin adverse effects. Nevertheless, the antifungal activity of 1000 μM TOH-containing emulgel need to be evaluated further in patients with cutaneous fungal infections, which we plan to investigate in the near future.

## Conclusions

The study confirmed positive effects of 1000 μM TOH-containing emulgel that exhibits antifungal activities and cosmeceutical benefits without causing skin irritation to human. The potential use of 1000 μM TOH-containing emulgel in patients with skin mycoses may be investigated in the future. Further, this study showed an innovative approach to utilize TOH-containing emulgel as a topical application for treating fungal infections as being in accordance with the growing use of emulgels in cosmeceutical and pharmacological products.

## Methods

### Materials

Tryptophol (TOH; C10H11NO,), voriconazole (VOZ,), absolute ethanol (EtOH), Tween^™^ 20, glycerin, propylene glycol, allantoin, phenoxyethanol, Acridine Orange/Ethidium Bromide (AO/EB), Sodium Lauryl Sulfate (SLS), and croton oil were purchased from Sigma-Aldrich (St. Louis, MO, USA). SEPIGEL 305^™^ was purchased from Seppic Inc. (Fairfield, NJ, USA). Triethanolamine was purchased from Dow Chemical (Thailand). Deionized (DI) water used in all experiments was produced from in-house Milli-Q^®^ IQ 7000 Ultrapure Water System (EMD Millipore, Bedford, MA, USA). Sabouraud Dextrose Agar (SDA) and Potato Dextrose Agar (PDA) were purchased from Oxoid Ltd. (Hampshire, UK). Roswell Park Memorial Institute 1640 medium (RPMI-1640), Fetal Bovine Serum (FBS), Penicillin-streptomycin (Pen-strep), Phosphate-Buffered Saline (PBS), and Trypsin-EDTA were purchased from Gibco™ (Grand Island, NY, USA). MTT (3-(4,5- dimethylthiazol-2-yl)-2,5-diphenyl tetrazolium bromide) was purchased from Bio Basic Inc. (Canada). CytoPainter MitoGreen (#ab176830) was purchased from Abcam. CytoTox 96^®^ Non-Radioactive Cytotoxicity assay for measuring the release of lactate dehydrogenase (LDH) was purchased from Promega (Madison, WI, USA). All the reagents used in this study were of high purity or HPLC grade.

### Preparation of TOH-containing emulgel formulation

The emulgel formulations were prepared with either tryptophol or voriconazole according to Kitisin *et al.*^9^. Optimization of all the emulgel formulations were performed by evaluating the effect of different concentrations of excipients based on visual changes in appearance, homogeneity, consistency, viscosity, stability, and any change in the physical characteristics prior to manufacturing the final formulations. The compositions of all the optimized emulgel formulae are provided in Table 3.

**Table 3:** Composition of different optimized emulgel formulations (% w/w).

Components	Emulgel formulation (% w/w)			
	Emulgel base	100 µM TOH	1000 µM TOH	1000 µM VOZ
1% EtOH as TOH diluent	10	–	–	–
TOH	–	10 (100 µM)	10 (1000 µM)	–
VOZ	–	–	–	10 (1000 µM)
SEPIGEL 305^™^	10	10	10	10
Triethanolamine	Q.s. to pH 5.5	Q.s. to pH 5.5	Q.s. to pH 5.5	Q.s. to pH 5.5
Tween^™^ 20	1	1	1	1
Glycerin	2	2	2	2
Propylene glycol	2	2	2	2
Allantoin	1	1	1	1
Phenoxyethanol	0.2	0.2	0.2	0.2
DI water	Q.s. to 100	Q.s. to 100	Q.s. to 100	Q.s. to 100
Total	100			

*EtOH* absolute ethanol, *TOH* Tryptophol, *VOZ* voriconazole, *SEPIGEL 305*^*™*^ polyacrylamide and C13-14 isoparaffin and laureth-7, *DI water* deionized water, *Q.s* quantity sufficient to adjust the pH to 5.5.

### Manufacturing process for emulgel

The emulgel base was manufactured with a constant mechanical stirring of the gel-based SEPIGEL 305^™^. TOH at 100 µM and 1000 µM or VOZ at 1000 µM were accurately prepared and added to the emulgel base and vigorously mixed until a smooth emulgel was formed^9^.

### Visual evaluation

The visual appearance of manufactured emulgel formulations were examined for their physical appearances including color, homogeneity, consistency, and phase separation^23^.

### Measurement of viscosity and pH

The viscosity of manufactured emulgel formulations was evaluated at room temperature using a Brookfield viscometer (Brookfield, Model programmable DV2, USA) with a volume sample of 0.5 g^9^. The formulated emulgel samples were left for equilibrium (30 min) at 25 °C. Measurements with the spindle were determined at 100 rpm for 10 min. The viscosity reading of each sample was recorded. Moreover, the pH of all manufactured emulgel formulations was measured by a digital pH meter (Thermo Fisher Scientific, Waltham, MA, USA)^23^. Prior measurement, pH meter was calibrated using standard pH buffers at 4, 7, and 10. Then, the pH for all the emulgel formulations was recorded. The pH meter probe was washed after each measurement with DI water and 70% (*v/v*) EtOH. Data were analyzed in tripiclate.

### Measurement of spreadability

The spreadability of manufactured emulgel formulations was investigated to determine the competence of each emulgel formulation, which is able to spread evenly after smeared on the affected skin^24^. To measure the spreadability of emulgel samples, 1 g of each formulated emulgel was placed onto the ground fixed glass plate and covered with another glass plate and placed on a wooden block. A definite load of about 20 g was then placed on the top of the slides for 1 min to provid the basis for the slip and drag characteristics of the emulgel. The time in seconds required to separate the two slides was recorded and analyzed in triplicate. The spreadability was calculated using the following equation: S = m * l/t; where S stands for spreadability, m stands for weight tied on upper slide; l is the length of glass slides; t, the time (s) requires to separate the slides completely^25^.

### Measurement of stability

Physical stability studies were investigated for all the formulations using using centrifugation and temperature cycle tests^9^. For centrifugation test, approximately 10 g of sample was centrifuged at 9000 rpm for 30 min. In addition, 2 g of each prepared emulgel samples was subject to a temperature cycle test by storing in 24 h/a cycle at − 4 °C (8 h) and 40 °C (16 h) for 10 cycles. After cooling-heating and freeze-thaw, the emulgel samples were evaluated for stability changes. All measurements were performed in triplicate. Physical stability of these emulgels formulations was evaluated at the end of these tests.

### Fungal strain and growth condition

*Scedosporium apiospermum* (CBS 117410), *S. boydii* (CBS 120157), and *Lomentospora prolificans* (CM324) used in this study were kindly provided by Dr. Ana Alastruey-Izquierdo (Servicio de Micologίa, Instituto de Salud. Carlos III, Madrid, Spain)^9^. *Aspergillus fumigatus* (CL-262; an azole resistant clinical strain carrying the M220I mutation in Cyp51A gene) was kindly provided by Asst. Prof. Dr. Marut Tangwattanachuleeporn (Faculty of Allied Health Sciences, Burapha University, Chonburi, Thailand)^10^. *Bipolaris maydis* was kindly provided by Dr. Songbai Zhang, (College of Agriculture, Yangtze University, Hubei, China)^26^. *Microsporum canis*^11^, *M. gypseum*^12^, *Trichophyton tonsurans*^11^, *Talaromyces marneffei*^13^, *Mucor* spp.^13^, *Candida tropicalis* (unpublished data), *Fonsecaea pedrosoi* (unpublished data), and *Rhizopus* spp. (unpublished data) used in this study were clinical or environmental isolates that previously collected and identified from our laboratory.

*A. fumigatus* (AF293; ATCC MYA-4609, CBS 101355; an azole sensitive strain), *Candida albicans* (ATCC 90028; an azole sensitive strain), *C. albicans* (ATCC 96901; an azole resistant strain), *C. dubliniensis* (ATCC MYA-646, CBS 7987), *T. mentagrophytes* (ATCC MYA-4439), *T. rubrum* (ATCC MYA-4438), *T. verrucosum* (ATCC 42898), *Epidermophyton floccosum* (ATCC 52066), *Acremonium recifei* (ATCC 36328), *Exophiala dermatitidis* (ATCC 76204), *Sporothrix schenckii* (ATCC 58251), *Malassezia furfur* (ATCC 12078), *T. asahii* (ATCC 90039), *Cryptococcus neoformans* (ATCC 32045), *C. gattii* (ATCC 56992), *Lodderomyces elongisporus* (ATCC 11503), and *Beauveria bassiana* (ATCC 74040) were provided by the American Type Culture Collection (ATCC) (Manassas, VA, USA). Fungal strains were cultured in SDA for yeasts at 37 ± 1 °C and PDA for molds at 25 ± 1 °C.

### In-vitro antifungal activity studies

*In-vitro* antifungal activity studies of manufactured emulgel formulations were evaluated using the agar spot assay according to Horváth *et al.*^27^ for yeasts and according to Muangkaew *et al.*^28^ for filamentous fungi with some modifications. Briftly, ten µl of each fungal conidia at concentration about about 5 × 10^6^ conidia/ml were mixed with 10 µl of each manufactured emulgel formulations (TOH at 100 or 1000μM or VOZ at 1000 μM) and spotted on the surface of SDA plates. Then, the plates were incubated at 37 ± 1 °C for 14 days. The colony diameter (mm) were measured in millimeters using a vernier caliper^4,9^. Uninoculated SDA plate was used as a negative control. The experiment was performed in triplicate.

### Cell culture, viability, and cytotoxicity

Human foreskin fibroblasts (HFF-1) cell line (ATCC #SCRC-1041™) was used to investigate cell viability and cytotoxicity after TOH treatments. HFF-1 cells were cultured in RPMI-1640 supplemented with 10% FBS and 100 U/ml of Pen-strep and incubated under humidified conditions with 5% CO_2_ at 37 °C. Measurements of cell viability and cytoxicity after TOH treatments were carried using MTT^9^, CytoPainter MitoGreen, LDH^9^, and dual AO/EB staining assays. As previously described, HFF-1 were seeded at a concentration of 1 × 10^5^ cells/ml in a 96 well plate and after 24 h were treated with 100 μl of TOH at 100 or 1000μM or VOZ at 1000 μM or with absolute EtOH and RPMI 1640 at 1% (v/v) as diluent controls. Untreated HFF-1 cells were used as negative controls (100% viable). After incubation for 24 h, 10 μl of 0.5 μg/μl MTT solution or 50 μl of CytoTox 96^®^ reagent were added and measured spectrophotometrically at 540 nm or 490 nm, respectively (Sunrise Tecan, Grödig, Austria). To label cell viability and toxicity, treated HFF-1 cells were stained with 100 μl of MitoGreen indicator or 25 μl of 100 μg/μl AO/EB solution, respectively. Cellular morphology and apoptotic cell numbers were examined by a Zeiss Axio Imager fluorescence microscope (ZEISS, Germany). All of the experiments were performed in triplicate.

### In vivo evaluation of the emulgel formulations on mouse skin irritation

Female BALB/c mice were purchased from Nomura Siam International Co, Ltd. (Thailand) and used to evaluate skin irritation of manufactured emulgel formulations. Each group included 5 animals (aged 6-8-week-old, body weight: 20–25 g) The skin irritation mouse model was produced and validated as described previously with some modification^22^. Briefly, all mice were topically treated with various concentrations of manufactured emulgel formulations (including emulgel base, 1000 µM of TOH or VOZ containing emulgels) that do not cause necrosis of the epidermis. Five percentage of SLS and croton oil were used as irritants. Commercial shielding cream was applied as controls. For single irritation, 15 μl of the irritants were put on the 8 mm Finn Chambers on Scanpor tape (Epitest Ltd., Finland), which were then applied on the dorsal skin of the animals. Polyurethane film (3M™ Tegaderm™, NY, USA) was used to fix them and Finn Chambers were then removed after 24 h post-application. For cumulative irritation, the procedure was repeated once a day for 1 and 2 weeks. After removing the Finn Chambers, mice were sacrificed, and skin samples were collected. The specimens were fixed in 10% buffered formalin and processed for haematoxylin and eosin (H&E) stain. All skin sections were examined with a light microscope (BH-2, Olympus, Japan). All animal experiments were conducted in accordance with the Animals for Scientific Purposes Act, B.E. 2558 (A.D. 2015), Thailand. Experiments on mice were approved by Institutional Animal Care and Use Committee (IACUC) of the University of Phayao, Thailand (approval number: UP-AE64-01-04-017).

### In vivo evaluation of the emulgel formulations on human skin irritation

To evaluate the sensitization potential of manufactured emulgel formulations toward human skin, a skin irritation test was performed by 30 healthy volunteers^29^. A single irritation test of the manufactured emulgel formulations (emulgel base, 1000 µM of TOH or VOZ containing emulgels) were put on the 8 mm Finn Chambers on the back of arm skin of the volunteers and fixed with polyurethane film. Commercial shielding cream was applied as controls. Finn Chambers were then removed after 24 h post-application. Treated areas of volunteer skin were observed immediately and 72 h after removing Finn Chambers. All *in vivo* studies with healthy volunteers are complied with the 2013 Declaration of Helsinki and was approved Ethics Committee (EC) at the Faculty of Tropical Medicine, Mahidol University, Thailand (approval number: MUTM 2022-036-01). Written informed consent was obtained from all the participants.

### In vivo evaluation of the emulgel formulations on human skin hydration, trans-epidermal water loss (TEWL), and skin color

To evaluate the effects of manufactured emulgel formulations on human skin physiological functions including skin hydration and trans-epidermal water loss (TEWL), treated skins of 30 healthy volunteers with manufactured emulgel formulations were investigated using DermaLab^®^ with a hydration probe and a TEWL probe (Cortex Technology, Hadsund, Denmark)^30^. After manufactured emulgel treatments, measurements of the skin hydration and TEWL were performed in 2 × 2 cm squares on the middle forearms, next to each other, with an interval of 5 s between each measurement. Three measurements were taken in each square.

### Statistical analysis

All the data was statistically analyzed using GraphPad Prism, version 6.05, (GraphPad Software, San Diego, CA, USA). All experiments were performed in triplicate. Data were expressed by mean ± SD. A *P*-value of < 0.05 was considered as statistically significant using an independent *t*-test or analyses of variance (ANOVA) followed by Tukey’s multiple comparison test.

### Institutional review board statement

All animal experiments were conducted in accordance with the Animals for Scientific Purposes Act, B.E. 2558 (A.D. 2015), Thailand. Experiments on mice were approved by Institutional Animal Care and Use Committee (IACUC) of the University of Phayao, Thailand (approval number: UP-AE64-01-04-017). Human studies were approved by Ethics Committee (EC) at the Faculty of Tropical Medicine, Mahidol University, Thailand (approval number: MUTM 2022-036-01).

### Informed consent

Informed consent was obtained from all human subjects involved in the study.

## Data Availability

The data collected and analyzed in the current study are available from the corresponding authors on reasonable request.
